# Dynamic Response and Service Life of Tunnel Bottom Structure Considering Hydro-Mechanical Coupling Effect under the Condition of Bedrock Softening

**DOI:** 10.3390/ma15186496

**Published:** 2022-09-19

**Authors:** Dengke Wang, Jianjun Luo, Feilong Li, Guanqing Wang, Lei Wang, Jie Su, Zhen Gao, Kunyao Yin

**Affiliations:** 1 Key Laboratory for Urban Underground Engineering of Ministry of Education, Beijing Jiaotong University, Beijing 100044, China; 2 Beijing Mass Transit Railway Operation Corp. Ltd., Beijing 100044, China; 3 Henan Yellow River Bureau Kaifeng Yellow River Bureau, Kaifeng 475004, China

**Keywords:** heavy-duty railway tunnel, concrete structure at tunnel bottom, hydro-mechanical coupling, bedrock softening, dynamic response, service life

## Abstract

Due to the long-term coupling effect of a train load and groundwater, the surrounding rock at the tunnel bottom will soften in a certain range and the mechanical parameters of the surrounding rock will decrease, causing the uneven distribution of the confining pressure at the tunnel bottom and affecting the base concrete structure service life. In this research, the method of combining field tests and numerical simulation is adopted, and the vertical displacement, vertical acceleration, and maximum and minimum principal stresses are used as evaluation indicators. The dynamic response law of the base structure with the softened surrounding rock of the heavy-duty train is analyzed, and the Miner linear cumulative damage theory is introduced to obtain the service life of the tunnel bottom structure under different softening conditions. The results show that with the decrease in the softening coefficient and the increase in the softening thickness of the bedrock, the displacement, acceleration, and principal stress response indexes of the structure increase by varying degrees, and the service life of the base structure decreases almost linearly. The maximum vertical displacement, acceleration, and tensile stress are located directly below the track, and the maximum compressive stress is located at the connection between the inverted arch and the side wall. According to the predicted value of the service life, the reliability of the base structure is divided into four levels: safety, warning, danger, and serious danger.

## 1. Introduction

With the increase in the service time of a tunnel, the bedrock at the bottom of the tunnel will be softened and damaged in a certain range and to different degrees with the coupling action of factors such as the train vibration load and groundwater [1,2,3]. The softening of the bedrock reduces its mechanical properties, forming unfavorable basic conditions at the bottom of the tunnel, affecting the safety and stability of the tunnel bottom structure [4]. Compared with ordinary railways, heavy-haul railways are more prone to serious tunnel damage due to the characteristics of heavy axles, large density, and large transportation volume. According to incomplete statistics, the damage rate of heavy-haul railways in China is 76.83%, which is 2.5 times that of general railway tunnels [5], and this damage is mainly concentrated at the bottom of the tunnel. For example, the basement settlement of the Changliangshan Tunnel of the Shuohuang railway reached 1.5 cm [6]. After the bedrock softens, if it is not treated in time, damage such as basement voids, mud-pumping, and inverted arch cracking will gradually form, which will seriously affect driving safety. Therefore, it is of great significance to study the dynamic response of the bedrock softening of the tunnel base structure with the coupling action of a train load and groundwater.

So far, some research has been carried out on the dynamic response of tunnels under the action of hydro-mechanical coupling. Mandal et al. [7] and Jeon et al. [8] have reported that the tunnel structure will be strongly squeezed by the large tectonic stress when the tunnel is located in the weak surrounding rock. Nejati et al. [9] took Tehran Metro Line 4 as an example, applied the dynamic load to the tunnel as a point load, and studied the ground vibration caused by the train vibration. Wang et al. [10] compared and analyzed the dynamic response and fatigue life of a tunnel base structure with water and without water through on-site monitoring and numerical simulation, revealing the adverse effects of water damage on the tunnel structure. Li et al. [11] analyzed the degradation range and depth of the surrounding rock at the bottom of a heavy-duty railway tunnel with different axle loads and the surrounding rock conditions by combining the method of laboratory testing and discrete element simulation, and they obtained 20 cm as the maximum degradation depth of the surrounding rock. Andersen et al. [12] tried to compare the response differences between the 2D model and the 3D model and concluded that the 3D model is more accurate for the absolute prediction of train-induced vibration, while the 2D model is only qualitatively feasible. Song et al. [13] considered the coupling of soil, water, and air, and analyzed in detail the dynamic response caused by the unit vertical harmonic point load acting on the inverted arch of the tunnel. Auersch [14] noted that the effect of train speed is always combined with the effect of reduced soil stiffness. Very soft soils result in lower train critical speeds and greater vibration of the track and ground compared to normal rigid soils. Yuan et al. [15] studied the effects of soil permeability and tunnel–soil interface permeability on soil displacement and pore pressure response. Takemiya et al. [16] proposed a simulation model to study the relationship between train vibration and train speed under the track. Pan et al. [17] established a train–tunnel–soil finite element model based on the multi-body dynamics theory and analyzed the dynamic response of the soil around a tunnel for a single operating condition and four typical encounter conditions.

The above scholars [7,8,9,10,11,12,13,14,15,16,17] have conducted a large number of related studies on the dynamic response of tunnel structure and soil under train vibration, However, they did not take into account the effects of bedrock softening. Bedrock softening can make the stress field around the tunnel structure more complex and more difficult to analyze [18]. Moreover, under the repeated (cyclic) load of long-term freight or heavy-duty trains, the phenomenon of mud-pumping will also form [19,20,21]. Hansen et al. [22] used a shaking table to simulate the degradation mechanism of roadbeds and obtained that the softening damage of surrounding rock under the action of hydrodynamic pressure can be divided into two categories: microstructure erosion and strength attenuation. Ma et al. [23,24] studied the variation law of the vibration acceleration and dynamic stress of a tunnel base structure under intact, damaged, and repaired conditions through on-site monitoring. Indraratna et al. [25] through a series of undrained cyclic triaxial tests, found that the softening of surrounding rock is due to the upward migration of water and fine particles in the soil, which eventually leads to the softening and fluidization of the uppermost part of the soil sample. Li et al. [26,27] studied the evolution law of the voiding of surrounding rock with different soil qualities (cohesive soil, pebble soil, and sandy soil) for a tunnel base using field monitoring and model tests, and they found that the cohesive soil was most affected by the train load and groundwater. Chai [28] used the finite difference software FLAC3D to study the dynamic response and structural damage of a tunnel base structure with different degrees of bedrock softening.

In general, some researchers [7,8,9,10,11,12,13,14,15,16,17] have made some progress in the study of the dynamic response of railway tunnel base structures under ideal conditions, but they have ignored the effect of bedrock softening. In order to consider the influence of bedrock softening, scholars have conducted some studies through laboratory experiments [19,20,21,22,25] and numerical simulations [23,24,26,27,28], but these studies have not considered groundwater. Therefore, this paper combines groundwater and bedrock softening, and establishes a three-dimensional numerical model considering train load–tunnel–groundwater–surrounding rock–bedrock softening. Taking the vertical displacement, vertical acceleration, maximum principal stress, and minimum principal stress as evaluation indicators, the dynamic response characteristics of water-rich tunnel basement structures under the condition of bedrock softening are studied systematically. In addition, the linear fatigue cumulative damage theory is introduced to study the long-term performance of the tunnel bottom structure with the softening of the bedrock and predict its service life.

## 2. Field Measurement

### 2.1. Survey Point Engineering Overview

**Figure 1:**
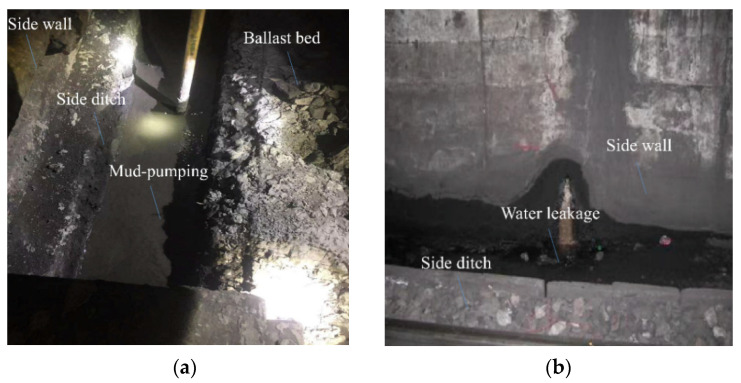
Main damage to heavy-duty railway tunnels: (**a**) Mud-pumping; (**b**) Leakage of lining.

**Figure 2:**
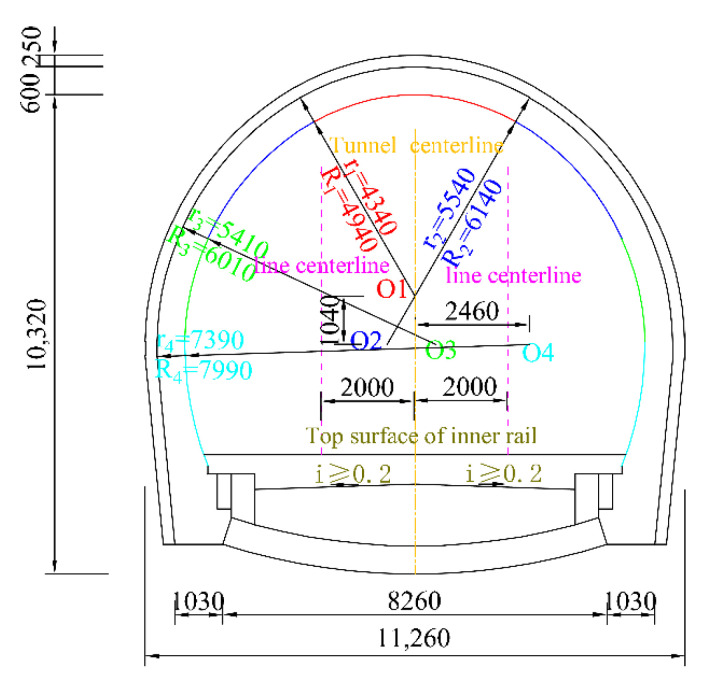
Design lining sectional drawing of grade-V surrounding rock (units: mm).

The measuring point tunnel is located in Shanxi. The lithology of the tunnel is relatively complex, the cross-bedding and fine-bedding are generally developed, and the interlayer bonding strength is low. In particular, the thin mudstone and shale sandwiched by sandstone can be peeled off very easily. The tunnel is mainly composed of feldspar sandstone, mudstone, and sandy mudstone. The hydrogeological conditions are complex, and the surface water and groundwater are well developed and in a recharge relationship. According to the on-site investigation, different types of damage have appeared in the tunnel since its opening and operation, among which the most serious types are mud-pumping and lining leakage (Figure 1). The tunnel is a single-hole double-track heavy-duty railway tunnel. The left line shows a heavy-duty line, and the right line shows an empty-carriage line. Figure 2 shows the design lining section of the grade-V surrounding rock.

### 2.2. Sensor Layout

**Figure 3:**
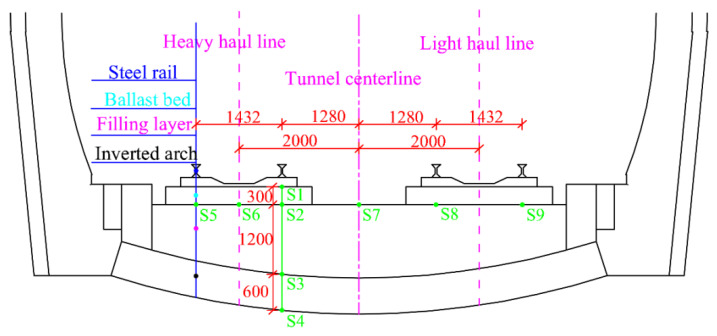
Schematic diagram of field test monitoring points (units: mm).

To understand the vertical propagation and lateral distribution of the train dynamic load on the tunnel base, the sensors are arranged as shown in Figure 3. As the underside of the heavy-duty line is the most affected by the dynamic load of the train [29,30], part of the sensor is buried directly under the heavy-duty line track, and the rest is buried on the surface of the filling layer. The section of the water-rich tunnel is selected for on-site testing, and the Digital Signal Processing (DSP) vibration testing system is selected for the on-site monitoring equipment. A fiber grating stress sensor with a range of 0.5 MPa is selected for the monitoring instrument. At the site, the main monitoring target is the vertical dynamic stress when a heavy-duty train with a 27 t axle load passes through the monitoring section at a speed of 80 km/h.

## 3. Numerical Simulation

### 3.1. Numerical Model Establishment

**Figure 4:**
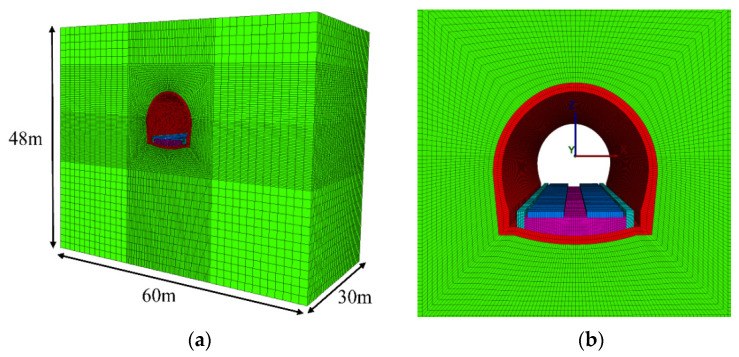
Numerical computation model: (**a**) Overall computational model; (**b**) Local enlarged drawing.

The finite difference software FLAC3D is used for three-dimensional numerical simulation, and the numerical model shown in Figure 4 is established. The model is taken as 30 m along the tunnel axis, which is about the length of two carriages of the train. The height is taken from the tunnel axis upward to the ground surface, and the width is taken as three times the tunnel diameter downward, which is about 30 m. The width is taken as three times the tunnel diameter from both sides of the tunnel axis, which is about 30 m. The overall size of the model is (X direction) 60 m × (Y direction) 30 m × (Z direction) 48 m. In this study, the tunnel structure and the surrounding rock are simulated by solid elements. To accurately describe the wave propagation in the numerical model, the size of the model element should be smaller than 1/10–1/8 of the wavelength corresponding to the highest frequency in the input wave during meshing [31].

Referring to the railway tunnel design specification [32] and the tunnel design data, the physical and mechanical parameters of the tunnel structure and the surrounding strata in the numerical model are shown in Table 1. The internal friction angle of the surrounding rock is 25°, the cohesion is 55 kPa, the permeability coefficient is 1.078 × 10^−6^ m/s, and the porosity is 0.45. The Mohr–Coulomb model is used for the static calculation of the water-rich formation, and the Byrne model is used for the dynamic calculation. The lining of the tunnel structure, the track bed, the filling layer, and the inverted arch all adopt the linear elastic model. The model boundary conditions are static boundary conditions to effectively absorb the body waves on the model boundary and reduce the influence of the boundary on the calculation results. The damping in the dynamic analysis adopts Rayleigh damping.

In order to simulate the process of the accumulation of pore water pressure in the soil at the bottom of the tunnel until the soil liquefies under dynamic action, the Byrne model [33] is used in the coupling calculation of dynamic calculation and seepage. The Byrne model is a further simplification of the Martin–Finn–Seed model [34,35,36], which simplifies the calculation of plastic volumetric strain increments $∆\varepsilon_{vd}$. Specifically, it can be calculated according to the following two formulas:

(1) $$∆\mu ={\bar{E}}_{r}∆\varepsilon_{vd},$$

(2) $$\frac{∆\varepsilon_{vd}}{\gamma }=C_{1}^{c}\exp\left(-C_{2}^{c}\frac{∆\varepsilon_{vd}}{\gamma }\right),$$

where ∆μ is the dynamic pore pressure increment, ${\bar{E}}_{r}$ is the one-dimensional elastic modulus of sand, and $C_{1}^{c}$ and $C_{2}^{c}$ are constants.

### 3.2. Application of Train Dynamic Load

At present, the excitation force function described in the literature [37,38,39,40,41] is widely used to simulate the load of a heavy-duty train. The exciting force function includes static load and dynamic load reflecting factors such as unevenness and the rail surface wave wear effect, and the superposition combination of the train wheelset force and the scattered transmission of rails and sleepers are considered. The influence of factors such as the train axle load and speed can be comprehensively discussed. The exciting force function is given as follows:

(3) $$P(t)=k_{1}k_{2}\left[p_{0}+p_{1}\sin(\omega_{1}t)+p_{2}\sin(\omega_{2}t)+p_{3}\sin(\omega_{3}t)\right],$$

where $p_{0}$ is the static load of the wheel, $p_{1}$, $p_{2}$, and $p_{3}$ are all vibration loads, $k_{1}$ is the superposition coefficient of the wheel–rail action, with a value range of 1.2–1.7, and $k_{2}$ is the dispersion coefficient of the wheel–rail action, with a value range of 0.6–0.9.

The unsprung mass of the train is denoted as $M_{0}$, and the corresponding train vibration amplitude is,

(4) $$p_{i}=M_{0}a_{i}\omega_{i}^{2}(i=1,2,3),$$

where $a_{i}$ is the typical vector height and $\omega_{i}$ is the circular frequency corresponding to the wavelength of the irregular vibration at the vehicle speed, and *i =* 1, 2, and 3 correspond to ①, ②, and ③ in Table 2, respectively. The formula for $\omega_{i}$ is,

(5) $$\omega_{i}=2\pi v/L_{i}(i=1,2,3),$$

where v is the train running speed and $L_{i}$ is the typical wavelength, and *i =* 1, 2, and 3 correspond to the control conditions ①, ②, and ③ in Table 2, respectively.

**Figure 5:**
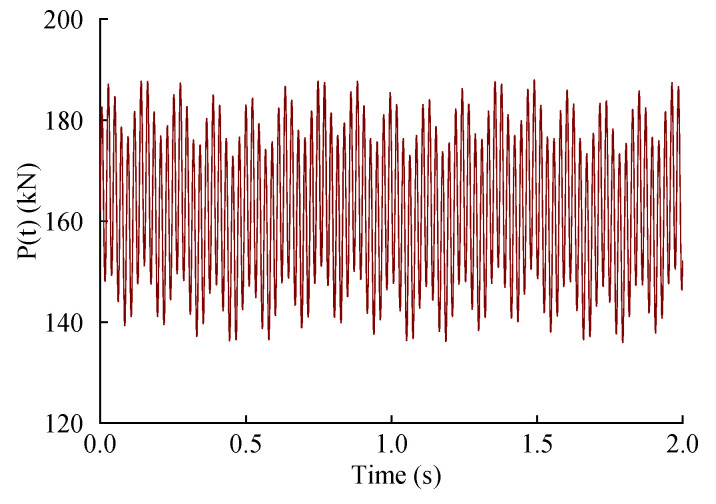
Time–history curve of train load.

When calculating the train load, the unilateral static wheel weight should be used, and the unsprung mass $M_{0}$ of the heavy-load train should be 1200 kg. According to Equation (3), under the 27 t axle load, the train load time–history curve when the train running speed is 80 km/h is as shown in Figure 5.

### 3.3. Simulation Conditions

For the softening depth, softening degree, and physical and mechanical parameters of surrounding rock, Forrest [42] pointed out that with the action of cyclic loading, a rock mass softens, and its parameters such as strength, elastic modulus, cohesion, and friction angle all have corresponding attenuations. Zhang [43] also gave the percentage of rock elastic modulus loss during freeze–thaw cycles and found that the elastic modulus of sandstone decreased by 87.5% and that of shale decreased by 45.1%. Liu [44] considered two influencing factors, the softening coefficient and the softening depth, where the softening coefficients were 1, 0.75, 0.5, and 0.25, and the softening depths were 1, 2, and 3 m. Ma [45] considered the simultaneous reduction of the elastic modulus, cohesion, and internal friction angle of the surrounding rock and defined the softening coefficients as 0.5 and 0.25. Wang [46] considered the simultaneous reduction of the strength and elastic modulus of the surrounding rock and studied six working conditions, including 100%, 70%, 50%, 40%, 30%, and 20%. Meng [47] selected the six softening coefficients of 0.5, 0.6, 0.7, 0.8, 0.9, and 1.0 to study the stability of surrounding rock for a roadway with different softening coefficients.

**Figure 6:**
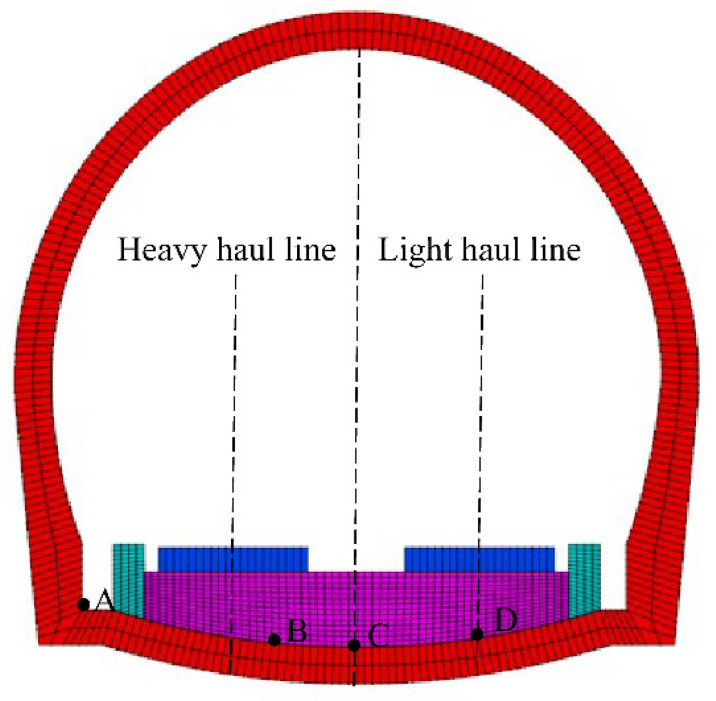
Schematic diagram of the monitoring point.

To summarize, in this research, the actual situation of the site and the research results of predecessors are combined, the softening degree and softening thickness are considered, and the softening layer covers the entire range of the inverted arch. Four points, A, B, C, and D, are selected as the analysis feature points to represent the connection between the side wall and the inverted arch, just below the track, the center of the inverted arch, and the center of the right line (Figure 6). The specific calculation conditions are as follows.

(1) The softening coefficient *K* is defined as the ratio of the elastic modulus of the surrounding rock after softening to the elastic modulus of the surrounding rock before softening. Taking the softening coefficient *K* as 1, 0.9, 0.8, 0.7, 0.6, 0.5, 0.3, and 0.1 and the softening thickness as 1 m, the dynamic response and the fatigue life of the tunnel bottom structure with the action of hydro-mechanical coupling are examined.
(2) The dynamic response and fatigue life of the tunnel bottom structure with hydro-mechanical coupling are analyzed for the eight different softening thicknesses of 0.5, 1.0, 1.5, 2.0, 2.5, 3.0, 4.0, and 5.0 m and the softening coefficient *K* of 0.8. The specific calculation conditions are shown in Table 3.

### 3.4. Numerical Simulation Verification

**Figure 7:**
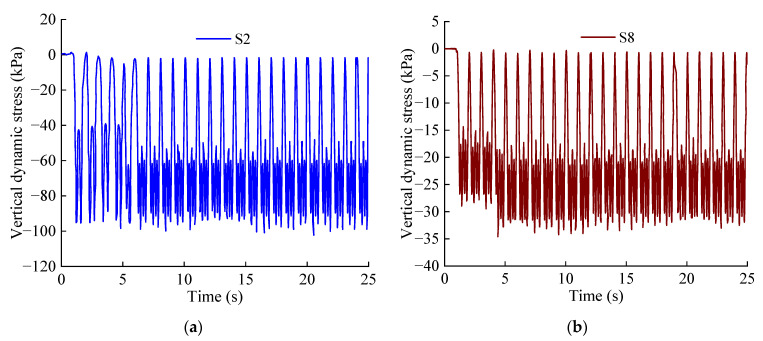
Time–history curve of vertical dynamic stress: (**a**) Measuring point S2; (**b**) Measuring point S8.

Figure 7 shows the vertical dynamic stress time–history curves of measuring points S2 and S8. When the train passes through the tunnel, the vertical dynamic stress distribution law of each measuring point is basically the same, and all of the points exhibit compressive stress. The vertical dynamic stress peak value of measuring point S2 is 102.3 kPa, and the vertical dynamic stress peak value of S8 is 34.6 kPa. It can be seen that the dynamic response of the area under the track of the heavy-load line is significantly higher than that of the area under the empty line.

**Figure 8:**
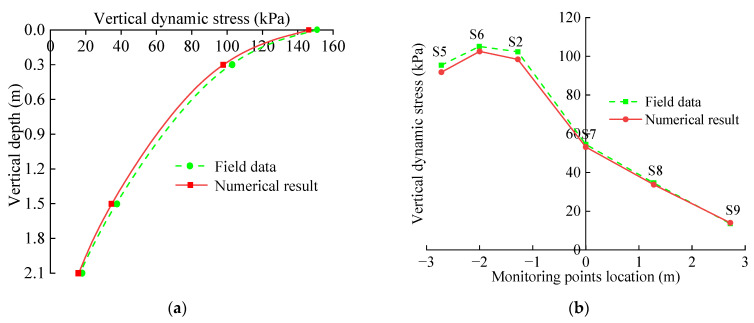
Comparison of dynamic field test and numerical simulation results: (**a**) Vertical attenuation contrast; (**b**) Horizontal distribution comparison.

To verify the reliability of the numerical model, the vertical dynamic stress peaks of the base measuring points S1–S9 with the action of the 27 t axis heavy load of the numerical simulation are extracted, as shown in Table 4, and the graph is drawn as shown in Figure 8. It can be seen from Table 4 that the dynamic stress peak value obtained with numerical calculation is not much different from the field measured results, and the overall deviation is between 2% and 9%, which is within 15% of the engineering requirements. There is a certain difference between the measured data and the simulation results in this research, which is due to the different action positions and action modes of field tests and numerical calculations. During field tests, the train load acts directly on the surface of the rail, and the dynamic load is affected by many factors such as the track irregularity value [48]. The numerical simulation acts directly on the surface of the track bed, and the train load is expressed by Formula (3). This also makes the dynamic stress time–history curves of field tests and numerical simulations different. However, the peak values of the two are relatively close, the attenuation curves of the vertical dynamic stress along the basement depth are basically the same (Figure 8a), and the horizontal distribution rules are basically the same (Figure 8b). This shows that the numerical model adopted in this research is reliable, which has been verified in the literature [10,29,49].

## 4. Results and Discussion

### 4.1. Displacement Response

**Figure 9:**
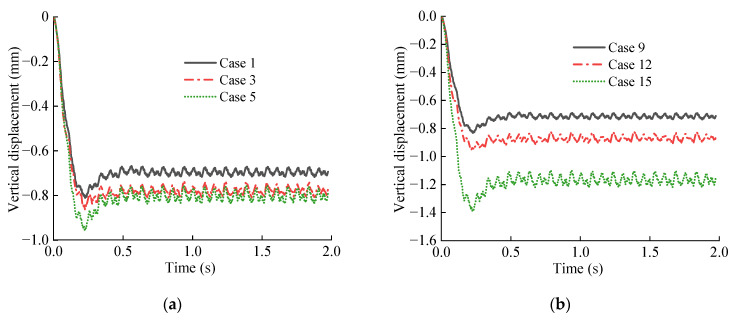
Vertical displacement time–history curve of measuring point B: (**a**) Different softening coefficients; (**b**) Different softening thickness.

Due to space limitations, Figure 9 only lists the displacement time–history curves of typical working conditions at point B directly below the track. It can be seen from Figure 9 that under different working conditions, the displacement time–history curve of the same part of the base structure has the same change rule, which is shown in the initial stage of train loading. The displacement increases rapidly to the peak value and then slightly rebounds to a certain level and changes periodically. In addition, with the decrease in the softening coefficient and the increase in the softening thickness, the magnitude of the vertical displacement and vibration amplitude of each part of the measuring point increase.

**Figure 10:**
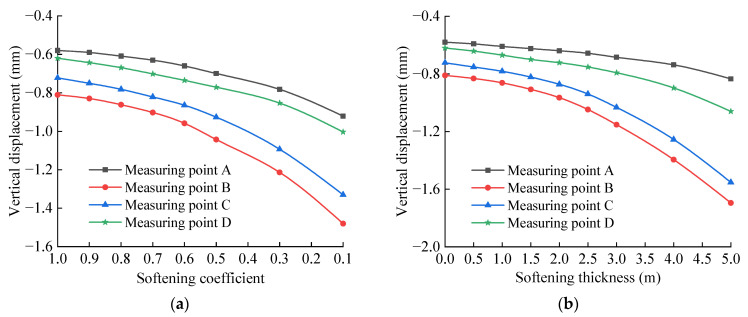
Vertical displacements peak change curve of base measuring point: (**a**) Relationship between vertical displacement and softening coefficient; (**b**) Relationship between vertical displacement and softening thickness.

The vertical displacement peak value of each measuring point of the base structure is extracted and drawn in the graph shown in Figure 10. It can be seen from Figure 10a that the vertical displacement of each characteristic point of the tunnel base increases with the decrease in the softening coefficient, and this increasing trend is obviously intensified as the value of the softening coefficient decreases. Specifically, when the softening coefficient value is 0.5, the vertical displacement increases significantly with the increase in softening degree. The maximum vertical displacement peak appears at measuring point B. When there is no softening layer, the vertical displacement of this position is –0.81 mm, and when the softening coefficient is 0.1, the vertical displacement of this position is –1.48 mm, and the latter value is 1.83 times the former value. It can be seen from Figure 10b that when the softening thickness of the bedrock increases from 0 to 5.0 m, the vertical displacement of each characteristic point of the basement structure increases continuously. Specifically, when the softening thickness value is 3.0 m, the increasing trend is obviously intensified. Among the feature points, the displacement of measurement point B is the largest, followed by point C, point D, and point A. When the softening thickness is 5.0 m, the displacement values are –1.69 mm, −1.55 mm, −1.06 mm, and −0.83 mm, respectively, which are 2.09, 2.15, 1.71, and 1.43 times that without the softening layer, respectively. It can be seen that the effect of the bedrock softening on the displacement response of the basement structure is obvious.

### 4.2. Acceleration Response

**Figure 11:**
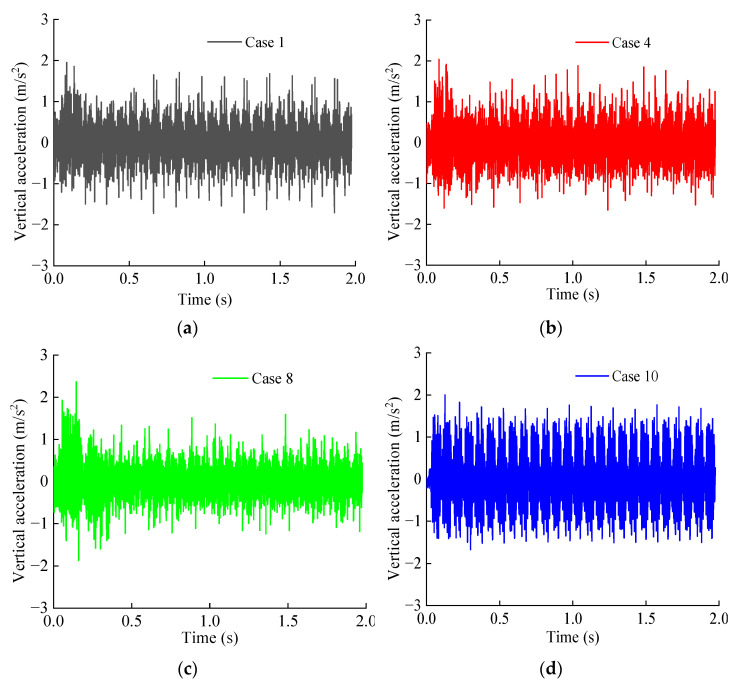

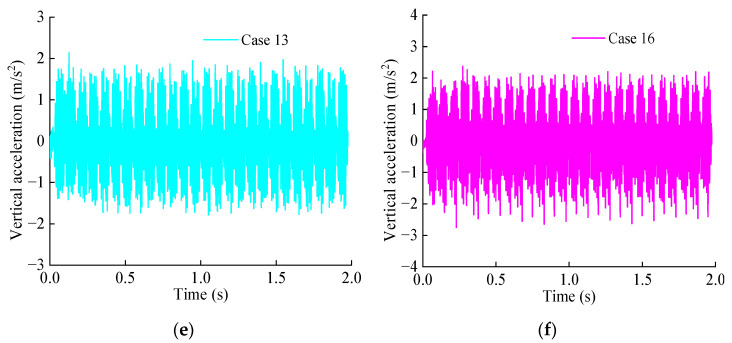
Vertical acceleration time–history curve of measuring point B: (**a**) Case 1; (**b**) Case 4; (**c**) Case 8; (**d**) Case 10; (**e**) Case 13; (**f**) Case 16.

Figure 11 shows the vertical acceleration time–history curve of typical operating conditions at point B directly below the track. It can be seen from Figure 11 that under different working conditions, the acceleration time–history curve of the same part of the base structure has the same variation law, and fluctuate up and down with the zero scale line as the center. In addition, with the decrease in the softening coefficient and the increase in the softening thickness, the magnitude of the vertical acceleration and the vibration amplitude of each part of the measuring point increase.

**Figure 12:**
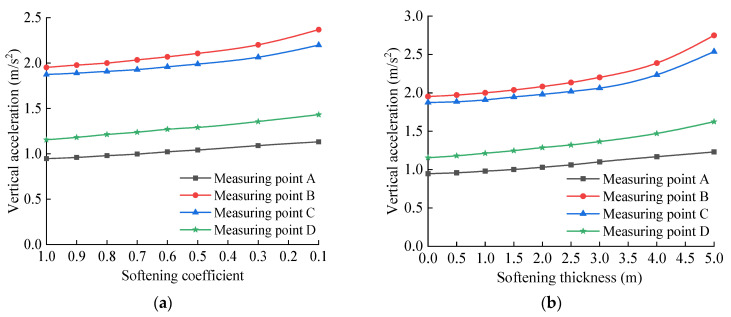
Vertical accelerations peak change curve of base measuring point: (**a**) Relationship between vertical displacement and softening coefficient; (**b**) Relationship between vertical displacement and softening thickness.

The vertical acceleration peak value of each measuring point of the base structure is extracted and drawn in the graph shown in Figure 12. It can be seen from Figure 12a that when the bedrock is not softened, the vertical acceleration peaks of measuring points A, B, C, and D are 0.95, 1.95, 1.87, and 1.16 m/s^2^, respectively. When the softening coefficient is 0.1, the vertical acceleration peaks of measuring points A, B, C, and D are 1.13, 2.37, 2.20, and 1.43 m/s^2^, and the latter values are 1.19, 1.22, 1.18, and 1.23 times the former values, respectively. The vertical acceleration of each measuring point of the base increases with the decrease in the softening coefficient, and this increasing trend is obviously intensified with the decrease in the softening coefficient value. Specifically, when the softening coefficient value is 0.5, the vertical acceleration increases obviously with the increase in the softening degree. It can be seen from Figure 12b that when the softening thickness of the bedrock increases from 0 to 5.0 m, the vertical acceleration of each measuring point of the basement structure increases continuously. In particular, when the softening thickness is 3.0 m, the increasing trend is obviously intensified. When the softening thickness is 5.0 m, the vertical acceleration peaks of measuring points A, B, C and D are 1.23, 2.75, 2.54, and 1.62 m/s^2^, respectively, which are 1.29, 1.41, 1.36, and 1.40 times the values when the bedrock is not softened. The softening of the tunnel bedrock does not change the overall distribution characteristics and time–history variation of the acceleration of the base structure, but it has a certain influence on the peak value of the acceleration of the structure, which strengthens the vibration response of the structure.

### 4.3. Principal Stress Response

**Figure 13:**
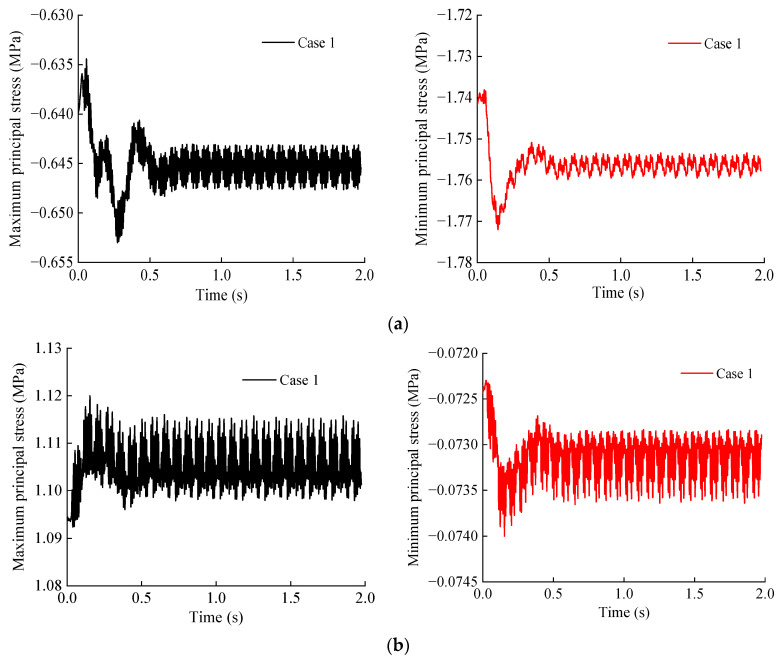

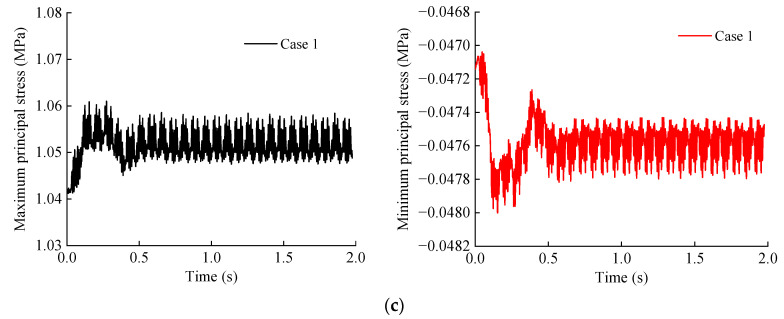
Time–history curve of principal stress at each measuring point of base structure: (**a**) Measuring point A; (**b**) Measuring point B; (**c**) Measuring point C.

Figure 13 shows the principal stress time–history curve under the typical working conditions (Case 1) of measuring points A, B, and C. It can be seen from Figure 13 that the principal stress time–history curves of different positions on the base structure are roughly the same, and the curves all show an increase at first and then slightly rebound to a certain level and then keep fluctuating up and down. The maximum tensile stress appears at the position just below the track (measurement point B), followed by the center of the inverted arch (measurement point C), and the maximum compressive stress appears at the connection between the side wall and the inverted arch (measurement point A).

**Figure 14:**
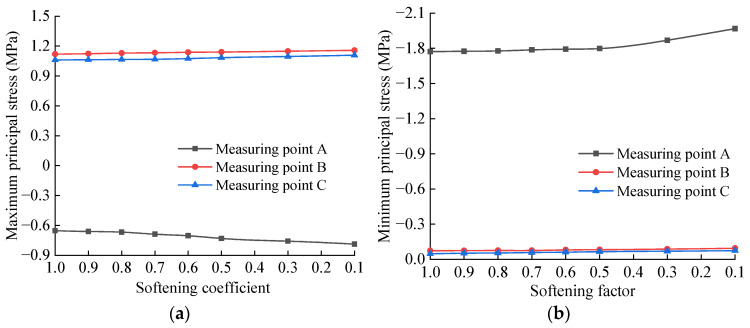
Variation curve of principal stress of base structure with different softening coefficients: (**a**) Maximum principal stress; (**b**) Minimum principal stress.

The principal stress peaks of each measuring point of the basement structure for different softening degrees of the bedrock are extracted, as shown in Table 5, and drawn on the graph shown in Figure 14. It can be seen from Table 5 and Figure 14 that the maximum and minimum principal stresses at each measuring point of the tunnel base increase continuously with the decrease in the softening coefficient. Specifically, after the softening coefficient value is 0.5, the principal stress increases obviously with the increase in the softening degree. When the softening coefficient of the bedrock is 0.1, the maximum tensile stress peak occurs at measuring point B, which is 1.158 MPa, followed by that of the measuring point C, which is 1.109 MPa, reaching 75.2% and 72.0% of the structural allowable tensile strength standard, respectively. The maximum compressive stress peak occurs at measuring point A, which is 1.968 MPa, which reaches 17.9% of the allowable compressive strength standard of the structure. It can be seen that the damage of the base invert is mainly controlled by the tensile strength of the structure, and the position of the invert directly below the track and the center of the invert are the most dangerous positions, which is consistent with the dynamic test results in the literature [50].

**Figure 15:**
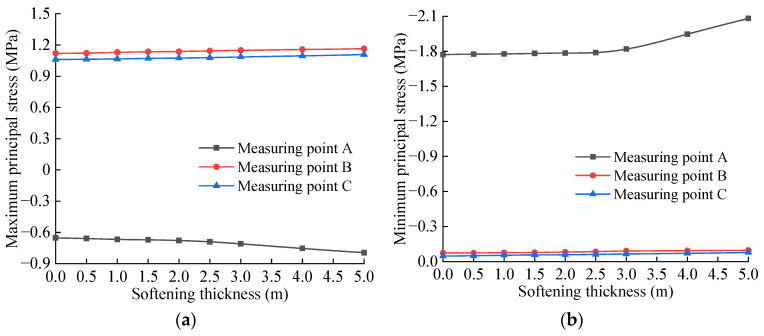
Variation curve of principal stress of base structure with different softening thickness: (**a**) Maximum principal stress; (**b**) Minimum principal stress.

The principal stress peaks of each measuring point of the basement structure for different softening thicknesses of the bedrock are extracted, as shown in Table 6, and drawn in the curve shown in Figure 15. It can be seen from Table 6 and Figure 15 that when the softened thickness of the bedrock increases from 0 to 5.0 m, the dynamic tensile stress and dynamic compressive stress of each measuring point of the inverted arch of the tunnel bottom increase continuously. In addition, this increasing trend is obviously intensified with the increase in the softening thickness. When the softening thickness of the bedrock is 5.0 m, the maximum tensile stress peak occurs at measuring point B, which is 1.164 MPa, followed by that of measuring point C, which is 1.108 MPa, reaching 75.6% and 71.9% of the structural allowable tensile strength standard, respectively. The maximum compressive stress peak occurs at measuring point A, which is 2.082 MPa, which reaches 18.9% of the structural allowable compressive strength standard. It can be seen that after the softening of the basement, the dynamic stress on the inverted arch structure at the bottom of the tunnel increases significantly, and this dynamic stress, especially the dynamic tensile stress, is very unfavorable to the stress on the inverted arch. From the calculation results, it can be observed that the bedrock is softened, and the basement structure is not damaged by the action of a single train load.

## 5. Influence of Bedrock Softening on the Long-Term Performance of Tunnel Structures

### 5.1. Principle and Numerical Realization of Concrete Fatigue Life

**Figure 16:**
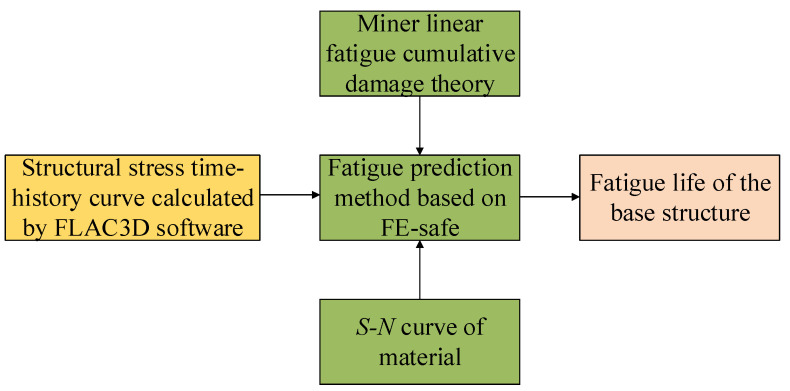
Fatigue life calculation process.

Although a single train vibration does not cause damage to the base structure, if the train vibration is regarded as a fatigue load acting on the structure, the base structure is subjected to thousands of repeated train vibration loads during the service of the tunnel. In addition, the softening of the bedrock causes the deterioration of the mechanical conditions of the structure, which may eventually lead to the fatigue failure of the structure. To evaluate the influence of bedrock softening on the service performance of the base structure, the fatigue analysis software FE-SAFE is used, and the most commonly used Miner linear cumulative damage theory is introduced to predict the fatigue life of the heavy-duty railway tunnel structure under different base conditions. The calculation steps are shown in Figure 16.

Ref. [51] pointed out that in the range of the random fatigue load and the high-cycle fatigue region, using the Miner linear fatigue cumulative damage criterion, the structural fatigue life analysis can accurately meet the engineering requirements, and this analysis has been widely used in engineering practice. In the fatigue calculation, it is first necessary to define the S–N curve of the material. The S–N curve in this research refers to the relationship between the stress amplitude and the fatigue life. The calculation formula of the stress amplitude is given as follows [52]:

(6) $$\sigma_{a}=\frac{\sigma_{\max}-\sigma_{\min}}{2},$$

where $\sigma_{a}$ is the stress amplitude, $\sigma_{\max}$ is the maximum stress value in the cycle, and $\sigma_{\min}$ is the minimum stress value in the cycle.

Considering the maximum and minimum stress levels of the structure, the fatigue life S–N curve used in the calculation model is expressed as follows:

(7) $$\mathrm{lg}N=16.67-16.76S_{\max}+5.17S_{\min},$$

(8) $$S_{\max}=\frac{\sigma_{\max}}{f},$$

(9) $$S_{\min}=\frac{\sigma_{\min}}{f},$$

where $S_{\max}$ is the maximum stress level, $S_{\min}$ is the minimum stress level, and f is the material ultimate strength.

Equation (7) can obtain the corresponding relationship between different stress amplitudes and the fatigue life. In addition, the influence of the average stress on the fatigue life is considered. The larger the average stress, the smaller the fatigue life of the corresponding structure. The expression for the mean stress is given as follows:

(10) $$\sigma_{m}=\frac{\sigma_{\max}+\sigma_{\min}}{2},$$

where $\sigma_{m}$ is the mean stress.

It can be seen from the abovementioned equation that the structural stress amplitude and the average stress level are the main factors affecting the fatigue life of the structure. The formula for calculating the service life *T* of the structure is,

(11) $$T=\frac{N}{365\times A},$$

where T is the service life of the structure, N is the fatigue life, which is generally expressed by lgN, and A is the daily number of trains in a tunnel, with the empirical value of 135.

### 5.2. Prediction of Concrete Fatigue Life

The dynamic response calculation results of different base softening coefficients and different base softening thicknesses are imported into the fatigue analysis software, and the fatigue calculation method and other fatigue calculation parameters are kept unchanged. The logarithmic fatigue life of the base structure under different working conditions is obtained, which is lgN. From the analysis described in Section 4.3, it can be seen that the most dangerous part of the base structure is the inverted arch just below the track (measurement point B), so this section only discusses the analysis of measurement point B. The service life of the structure with different softening coefficients is shown in Table 7. The service life *T* of the structure is calculated as follows:

$$\mathrm{Case}\;1:\;T=\frac{10^{6.667}}{365\times 135}=94.3\;a,\;\mathrm{Case}\;2:\;T=\frac{10^{6.616}}{365\times 135}=83.8\;a,$$

$$\mathrm{Case}\;3:\;T=\frac{10^{6.575}}{365\times 135}=76.3\;a,\;\mathrm{Case}\;4:\;T=\frac{10^{6.538}}{365\times 135}=70.0\;a,$$

$$\mathrm{Case}\;5:\;T=\frac{10^{6.426}}{365\times 135}=54.1\;a,\;\mathrm{Case}\;6:\;T=\frac{10^{6.326}}{365\times 135}=43.0\;a,$$

$$\mathrm{Case}\;7:\;T=\frac{10^{6.164}}{365\times 135}=29.6\;a,\;\mathrm{Case}\;8:\;T=\frac{10^{5.806}}{365\times 135}=13.0\;a.$$

Similarly, the service life of the base structure with different softening thicknesses can be obtained, as shown in Table 8.

**Figure 17:**
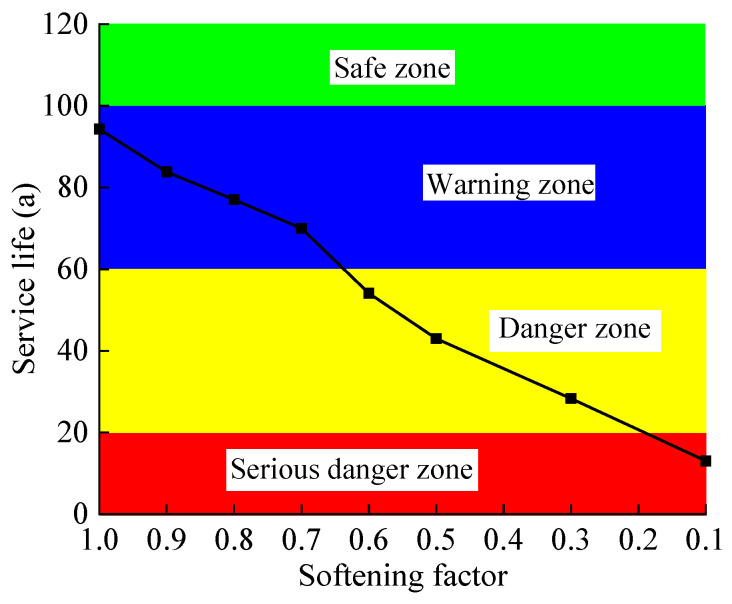
Service life of base structure with different softening coefficients.

**Figure 18:**
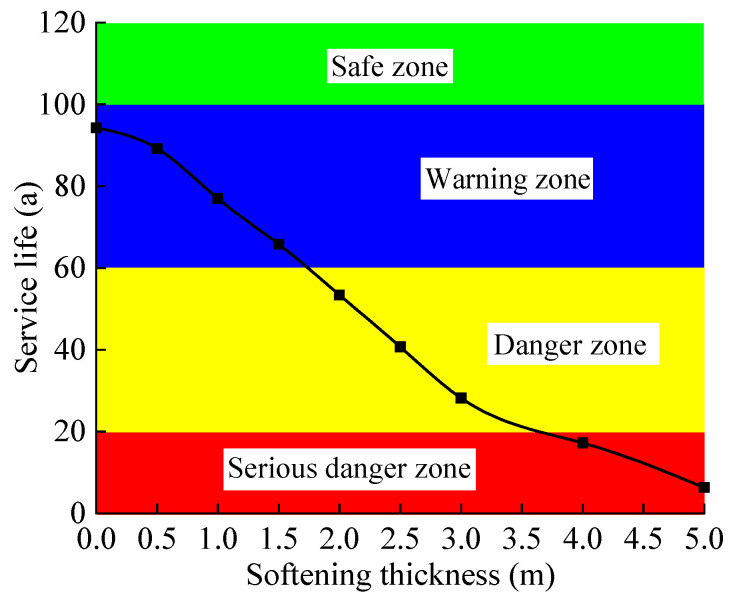
Service life of base structure with different softening thicknesses.

With reference to the relevant regulations on the reliability of tunnel structures in the “Unified Standard for Reliability Design of Railway Engineering Structures” (GB50126-94), the design reference period for tunnel structures is 100 years. The service life of the tunnel base inverted arch is divided into four grades. A service life of >100 years is the safe zone, which is indicated in green. A service life of 60 years < service life < 100 years is the warning zone, which is indicated in blue. A service life of 20 years < service life < 60 years is the danger zone, which is indicated by yellow. A service life of fewer than 20 years is a serious danger zone, which is indicated in red. According to the calculation of service life shown in Table 7 and Table 8, the trend diagram of the service life of the tunnel base structure under different softening conditions is given (Figure 17 and Figure 18). It can be seen from Figure 17 and Figure 18 that as the softening coefficient decreases and the softening thickness increases, the service life of the base inverted arch shows an almost linear decrease. When the bedrock is not softened but there is groundwater, the inverted arch structure cannot meet the requirements of the service life. When the softening coefficient of the bedrock is 0.1–0.3, the tunnel is in a serious danger zone; when the softening coefficient is 0.3–0.6, it is in a danger zone; and when the softening coefficient is 0.6–1.0, it is in a warning zone. When the softening thickness of the bedrock is 0–1.5 m, the tunnel is in a warning zone; when the softening thickness is 1.5–3 m, it is in a dangerous zone; and when the softening thickness is 3.0–5.0 m, it is in a serious danger zone. In particular, when the softening thickness is 5.0 m, the service life of the inverted arch of the tunnel is only 6.3 years, a decrease of 93.7%, which is close to fatigue failure. This shows that the softening of the bedrock has a significant effect on the service performance of the tunnel structure. It is recommended to adopt targeted remediation measures for the distribution areas of different service lives to ensure the safety of trains.

## 6. Conclusions

In this research, the method of combining a field test and numerical simulation is adopted. A three-dimensional model of train load–tunnel–surrounding rock–groundwater–bedrock softening is established. The dynamic response characteristics of the tunnel bottom structure with different softening degrees and different softening thicknesses of the bedrock are studied. Combined with the fatigue life prediction method, the influence degree of the bedrock softening on the fatigue life of the tunnel bottom structure is determined. The main conclusions are given as follows.

(1) The softening of the bedrock (softening degree and softening thickness) has a significant impact on the displacement response of the tunnel bottom structure. In particular, when the softening coefficient is 0.5 and the softening thickness is 3.0 m, the displacement response intensifies. When the softened thickness of the bedrock is 5.0 m, the vertical displacement is 2.09 times that without softening.
(2) The softening of the bedrock has little effect on the acceleration response of the tunnel bottom structure, but it still has a certain impact on the structural acceleration. After the bedrock softens, the acceleration of the measuring point is 1.29–1.41 times that of the unsoftened bedrock, which increases the vibration response of the structure.
(3) For different softening degrees and different softening thicknesses of the bedrock, the distribution law of the principal stress response of the tunnel bottom structure is similar. The maximum tensile stress appears just below the track, and the maximum compressive stress appears at the connection between the inverted arch and the side wall.
(4) A prediction method for the fatigue life of the base structure considering the softening of the bedrock is established, and the service life value of the inverted arch structure under different softening conditions is obtained. In addition, according to the predicted value, the reliability of the inverted arch is divided into four levels: safety zone, warning zone, danger zone, and serious danger zone.
(5) The on-site investigation shows that the tunnel has many places where mud-pumping occurs, indicating that some of the bedrock at the bottom of the tunnel have softened, lost, and formed voids. Furthermore, according to the field direct shear test, the mechanical parameters within 2 m of the soil at the bottom of the tunnel are reduced to varying degrees. It is comprehensively judged that the tunnel is in a danger zone, and corresponding measures should be taken immediately.

## Figures and Tables

**Table 1:** Physical and mechanical parameters.

Materials	Elastic Modulus *E* (GPa)	Poisson’s Ratio μ	Density ρ (kg/m^3^)
Grade-V surrounding rock	0.40	0.35	1850
Track bed	33.5	0.2	2700
Inverted filling	28.5	0.2	2300
Inverted arch	31.0	0.2	2600
Secondary lining	31.0	0.2	2600
Initial support	28.5	0.2	2500

**Table 2:** Management values for track geometric irregularities in the United Kingdom.

Control Conditions	Wavelength (m)	Versine (mm)
① Ride performance	50.00	16.00
	20.00	9.00
	10.00	5.00
② Dynamic additional load acting on the line	5.00	2.50
	2.00	0.60
	1.00	0.30
③ Corrugated wear	0.50	0.10
	0.05	0.005

**Table 3:** Calculation conditions.

Calculation Cases	Softening Coefficient	Softening Thickness (m)	Calculation Cases	Softening Coefficient	Softening Thickness (m)
Case 1	1	1.0	Case 9	0.8	0.5
Case 2	0.9	1.0	Case 10	0.8	1.0
Case 3	0.8	1.0	Case 11	0.8	1.5
Case 4	0.7	1.0	Case 12	0.8	2.0
Case 5	0.6	1.0	Case 13	0.8	2.5
Case 6	0.5	1.0	Case 14	0.8	3.0
Case 7	0.3	1.0	Case 15	0.8	4.0
Case 8	0.1	1.0	Case 16	0.8	5.0

**Table 4:** Peak values of vertical dynamic stress at different positions.

Measuring Point Location	Measured Dynamic Stress Peak Values (kPa)	Simulated Dynamic Stress Peak Values (kPa)	Deviation (%)
S1	150.8	145.3	3.76
S2	102.3	98.4	3.96
S3	36.0	33.7	6.87
S4	15.7	14.5	8.51
S5	95.4	91.8	3.91
S6	105.1	102.5	2.57
S7	54.5	53.1	2.62
S8	34.6	33.7	2.63
S9	13.6	14.0	2.91

**Table 5:** Maximum and minimum principal stress of base structure with different softening coefficients.

Softening Coefficient	Connection (MPa)		Directly below the Track (MPa)		Inverted Arch Center (MPa)	
	$\sigma_{1}$	$\sigma_{3}$	$\sigma_{1}$	$\sigma_{3}$	$\sigma_{1}$	$\sigma_{3}$
1.0	–0.653	–1.772	1.12	–0.074	1.061	–0.048
0.9	–0.661	–1.775	1.124	–0.075	1.063	–0.053
0.8	–0.667	–1.778	1.13	–0.076	1.066	–0.055
0.7	–0.688	–1.787	1.133	–0.076	1.068	–0.059
0.6	–0.704	–1.793	1.138	–0.081	1.075	–0.062
0.5	–0.731	–1.798	1.141	–0.083	1.084	–0.066
0.3	–0.758	–1.869	1.149	–0.088	1.096	–0.07
0.1	–0.787	–1.968	1.158	–0.095	1.109	–0.075

**Table 6:** Maximum and minimum principal stress of base structure with different softening thicknesses.

Softening Thickness (m)	Connection (MPa)		Directly below the Track (MPa)		Inverted Arch Center (MPa)	
	$\sigma_{1}$	$\sigma_{3}$	$\sigma_{1}$	$\sigma_{3}$	$\sigma_{1}$	$\sigma_{3}$
0	–0.653	–1.772	1.12	–0.074	1.061	–0.048
0.5	–0.659	–1.776	1.121	–0.074	1.063	–0.05
1.0	–0.667	–1.778	1.13	–0.076	1.066	–0.055
1.5	–0.671	–1.782	1.135	–0.078	1.072	–0.057
2.0	–0.689	–1.785	1.137	–0.082	1.075	–0.059
2.5	–0.695	–1.789	1.144	–0.085	1.078	–0.062
3.0	–0.709	–1.819	1.149	–0.092	1.086	–0.066
4.0	–0.762	–1.947	1.157	–0.093	1.095	–0.071
5.0	–0.795	–2.082	1.164	–0.097	1.108	–0.078

**Table 7:** Service lives of structures with different softening coefficients.

Case	Case 1	Case 2	Case 3	Case 4	Case 5	Case 6	Case 7	Case 8
lgN	6.667	6.616	6.579	6.538	6.426	6.326	6.164	5.806
T(a)	94.3	83.8	77.0	70.0	54.1	43.0	29.6	13.0

**Table 8:** Service lives of structures with different softening thicknesses.

Case	Case 9	Case10	Case 11	Case 12	Case 13	Case 14	Case 15	Case 16
lgN	6.643	6.579	6.511	6.420	6.302	6.143	5.927	5.493
T(a)	89.2	77.0	65.8	53.4	40.7	28.2	17.2	6.3

## Data Availability

Not applicable.
